# Systematics of the freshwater leech genus *Hirudinaria* Whitman, 1886 (Arhynchobdellida, Hirudinidae) from northeastern Thailand

**DOI:** 10.3897/zookeys.452.7528

**Published:** 2014-11-04

**Authors:** Jaruwan Tubtimon, Ekgachai Jeratthitikul, Chirasak Sutcharit, Bangon Kongim, Somsak Panha

**Affiliations:** 1 Animal Systematics Research Unit, Department of Biology, Faculty of Science, Chulalongkorn University, Bangkok 10330, Thailand; 2 Department of Biology, Faculty of Science, Mahasarakham University, Kantharawichai District, Mahasarakham 44150, Thailand

**Keywords:** Freshwater leeches, Hirudinea, karyotypes, morphology, COI, sanguivorous

## Abstract

In total, 435 specimens of the Southeast Asian freshwater leech species within the Hirudinidae family were collected from 17 locations of various types of aquatic habitats in northeastern Thailand. They were all morphologically placed within the genus *Hirudinaria* Whitman, 1886 and there were three distinct species: the common *Hirudinaria manillensis*, 78.2% of all collected specimens and at all 17 locations, *Hirudinaria javanica* at 20.3% of collected samples and from five locations and a rarer unidentified morphospecies (*Hirudinaria* sp.) with six samples from only two locations. The karyotypes of these three species were examined across their range in this study area for 38, 11 and 6 adult specimens of *Hirudinaria manillensis*, *Hirudinaria javanica* and *Hirudinaria* sp., respectively. This revealed different chromosome numbers among all three species, with *Hirudinaria javanica* having n = 13, 2n = 26, *Hirudinaria manillensis* lacked one small chromosome pair with n = 12, 2n = 24, and the unknown *Hirudinaria* sp. differed from any known *Hirudinaria* karyotypes in exhibiting a higher chromosome number (n = 14, 2n = 28) and a gradual change in size from large to small chromosomes. This suggests that the unknown *Hirudinaria* sp. is a new biological species. However, phylogenetic analysis based upon a 658 bp fragment of the cytochrome oxidase subunit I gene placed this unknown morphospecies within the *Hirudinaria manillensis* clade, perhaps then suggesting a recent sympatric speciation, although this requires further confirmation. Regardless, the chromosomes of all three species were asymmetric, most with telocentric elements. A distinct bi-armed chromosome marker was present on the first chromosome pair in *Hirudinaria javanica*, whilst it was on pairs 1, 2, 3 and 5 in *Hirudinaria manillensis*, and on pairs 3 and 5 for the unknown *Hirudinaria* sp.

## Introduction

The family Hirudinidae (Arhynchobdellida, Hirudiniformes) is comprised of mainly blood-sucking (sanguivorous) freshwater leeches, or medicinal leeches, although four terrestrial species are known. It includes approximately 60 hirudinids ranging across all continents, except for Antarctica, and from temperate to tropical regions (Elliott and Kutschera 2011). On the basis of the number of complete somites, the distance between the third and fourth pair of eyes, number of sensillae, position of the nephropore opening, and the presence or absence of auricular characters, the Hirudinidae family is divided into the two subfamilies of Hirudininae and Haemadipsinae. The Hirudininae, or buffalo leeches, contains 12 known species (Moore 1927) within six genera (*Dinobdella*, *Hirudinaria*, *Hirudo*, *Limnatis*, *Myxobdella* and *Whitmania*), and are distributed in temperate and tropical Asia, Africa and the Caribbean islands (Richardson 1969, Sket and Trontelj 2008, see Phillips and Siddall 2009 for alternative classification). The high species diversity and their wide geographic distribution make the hirudinid leeches attractive material for systematic and biogeographical studies. However, due to their conserved morphology, it is not easy to establish a reliable phylogenetic hypothesis for this group. There is only one recent published paper regarding their phylogenetic relationship that considered both morphological and molecular analyses and described a new species of the Asian buffalo leech, *Hirudinaria bpling* Phillips, 2012.

The genus *Hirudinaria* Whitman, 1886 consists of only three known species *Hirudinaria javanica* (Wahlberg, 1856), *Hirudinaria manillensis* (Lesson, 1842), and *Hirudinaria bpling* that are widely distributed over tropical South and Southeast Asia, being recorded from within Peninsular Malaysia, Thailand, Indo-China, Indonesia, Philippines, China, Myanmar, Bangladesh, India and Sri Lanka (Moore 1938, Lai and Chen 2010). Chromosomal data for hirudinid leeches have only been recorded for three species of *Hirudo* (Utevsky et al. 2009).

In this study, we examined the karyotypes of 38, 11 and 6 specimens of the three species (*Hirudinaria manillensis*, *Hirudinaria javanica*, and a third distinct and different morphospecies, *Hirudinaria* sp.) collected from across 17 locations in northeastern Thailand, representing 13.4%, 12% and 100% of the collected samples, respectively. Their systematic implications are then discussed in comparison with other previously reported hirudinid karyotypes. The phylogenetic analysis, based upon a 658 bp fragment of the cytochrome oxidase subunit I gene, was also conducted to clarify the systematics of all collected morphospecies.

## Materials and methods

Locality, co-ordination and sample size for all collected species are given in Table 1. Species identification of each specimen was made on the basis of Lesson (1842), Wahlberg (1856), Whitman (1886), Moore (1927), Richardson (1969), Klemm (1972) and Lai and Chen (2010). Voucher specimens were deposited in the Museum of Zoology, Chulalongkorn University, Bangkok, Thailand (CUMZ).

**Figure 1.**
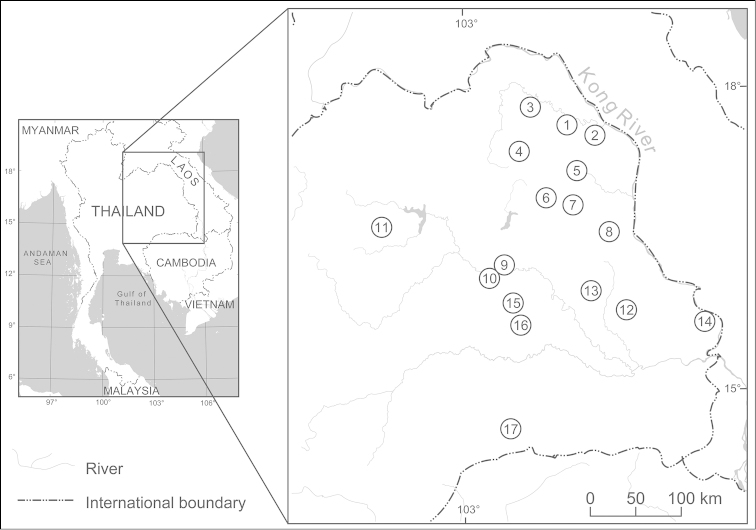
Map showing the locality of the sampling sites (collection of specimens from the genus *Hirudinaria*) in northeastern Thailand. Further details of sample numbers and locations are given in Table 1.

Freshwater leeches were collected from 17 localities in northeastern Thailand (Fig. 1 and Table 1) during invertebrate faunal surveys performed from April 2012 to February 2014. In total, 435 adult specimens were collected and examined. Specimens were photographed and kept alive in a glass aquarium in order to observe the body color pattern and other external morphological characteristics, plus any behavioral traits. Most specimens were relaxed in 10% (v/v) ethanol and then ﬁxed and kept in 95% (v/v) ethanol for further external and internal morphological studies. Some specimens were brought back alive to the laboratory for karyotypic analysis.

**Table 1.** Locality, co-ordination and sample size of each species used in the present study. Locality numbers refer to the localities shown in Figure 1.

No.	Locality	Coordinates	Number of specimens examined		
			*Hirudinaria javanica*	*Hirudinaria manillensis*	*Hirudinaria* sp.
1	Ban Donsala, Na Wa, Nakhon Phanom	17°34'27.22"N, 104°7'18.64"E	44	82	5
2	Ban Majang, Na Wa, Nakhon Phanom	17°36'53.4"N, 104°8'21.9"E	-	51	1
3	Ban Nongwang, Tao Ngoi, Sakon Nakhon	17°45'41.26"N, 103°44'42.00"E	9	4	-
4	Phang Khon, Sakon Nakhon	17°22'29.02"N, 103°40'26.81"E	-	2	-
5	Mueang, Sakon Nakhon	17°10'52.69"N, 104°7'50.94"E	-	2	-
6	Phu Phan, Sakon Nakhon	16°54'14.64"N, 103°54'7.50"E	-	6	-
7	Ban Janpen, Tao Ngoi, Sakon Nakhon	16°55'32.59"N, 104°10'9.31"E	16	1	-
8	Ban Nonghai, Khamcha-i, Mukdahan	16°34'53.92"N, 104°29'29.00"E	13	13	-
9	Khong Chai, Kalasin	16°15'44.76"N, 103°27'22.91"E	-	28	-
10	Ban Thatoom, Mueang, Mahasarakham	16°10'48.40"N, 103°26'59.30"E	-	4	-
11	Huai E-pong, Phu Wiang, Khon Kaen	16°43'51.30"N, 102°17'17.00"E	-	11	-
12	Tumbon Bung, Mueang Amnat Charoen	15°50'21.48"N, 104°27'33.95"E	-	30	-
13	Pa Tio, Yasothon	15°57'2.81"N, 104°25'12.78"E	-	3	-
14	Khemarat, Ubon Ratchathani	15°59'11.82"N, 105°8'20.53"E	-	26	-
15	Chaturaphak Phiman, Roi Et	15°49'59.77"N, 103°31'0.86"E	1	5	-
16	Kaset Wisai, Roi Et	15°39'13.70"N, 103°35'58.39"E	-	67	-
17	Huai Saneng Reservoir, Surin	14°47'14.70"N, 103°28'34.50"E	-	11	-

Jaws of some specimens were examined by scanning electron microscopy (SEM). The dried specimens were sputter coated with 35 nm of gold/palladium before being examined using a LEO/Zeiss DSM982 Geminifield emission scanning electron microscope located in the Scientific and Technological Research Equipment Centre, Chulalongkorn University.

Chromosome preparations were made from the testisac using hypotonic, fixation and air-drying techniques modified from Patterson and Burch (1978) and Kongim et al. (2013). Live leeches were injected with 0.1 mL of 0.1% (v/v) colchicine, left for 3–4 h and then dissected to remove the testisacs into 0.07% (w/v) KCl solution (hypotonic) for 30 min. Samples were then fixed in fresh Carnoy’s fixative (3:1 (v/v) absolute ethanol: glacial acetic acid). The testisacs were cut into small pieces in fresh Carnoy’s fixative and the separated cells were collected by centrifugation at 1,500 rpm for 10 min. The supernatant was removed and the cell pellet resuspended in 0.5 mL of fresh Carnoy’s fixative. Cell suspensions were dropped onto clean pre-heated (60 °C) glass slides, air-dried and stained in 4% (v/v) Giemsa solution for 10 min. Photomicrographs of 10 to 15 well-spread metaphase cells were measured for their relative length and centromeric index. Mitotic karyotypes were arranged and numbered for chromosome pairs.

For the molecular analysis, the total genomic DNA was extracted from a part of the wall-body muscle to avoid contamination from the host DNA, following the standard protocol of the DNeasy Blood & Tissue Kit (Qiagen Inc., Valencia, CA, USA). A fragment of the mitochondrial cytochrome oxidase subunit I (COI) gene was amplified using the primers LCO1490 (5’-GGT CAA CAA ATC ATA AAG ATA TTG G-3’) and HCO2198 (5’-TAA ACT TCA GGG TGA CCA AAA AAT CA-3’), which is the region used in animal DNA barcoding (Folmer et al. 1994). Polymerase chain reaction (PCR) of a 50 µL final volume using 20 µM of 2×Illustra hot starts master mix (GE Healthcare), plus 10 µM of each primer and about 10 ng of DNA temfig was performed in an eppendorf Mastercycler® pro S PCR thermal cycler with the following thermal cycling conditions: 3 min at 94 °C followed by 35 cycles of 1 min at 94 °C, 1 min at 45 °C and 150 s at 72 °C, before a final extension at 72 °C for 5 min. The PCR products were purified with a QIAquick PCR purification Kit (QIAGEN Inc.) before being commercially direct cycle-sequenced at Macrogen, Inc, Korea.

Sequence alignment and editing were performed using MEGA 6.06 (Tamura et al. 2013). The best-fit models of nucleotide substitution, as judged by the Akaike information criterion (AIC: Akaike 1974), were estimated using Kakusan4 (Tanabe 2007; with maximum likelihoods calculated in Treefinder, Jobb et al. 2004). The best-fit evolution model obtained was GTR+G. Phylogenetic trees based on maximum likelihood (ML) and Bayesian inference (BI) were constructed. The ML analysis was performed with Treefinder (Jobb et al. 2004), using the likelihood-ratchet method with 1000 bootstrap replicates. The BI tree was constructed using MrBayes v3.2.2 (Ronquist et al. 2012), which employs a Metropolis-coupled, Markov chain Monte Carlo (MC-MCMC) sampling approach. The BI analysis was run twice in parallel for one million generations (with default heating values), starting with a random tree, and trees were sampled every 100 generations. The remaining trees, after discarding 25% of ‘‘burn-in’’ samples, were used for calculation of the bipartition posterior probability (Ronquist et al. 2012). Tree topologies with bootstrap values of 70% or greater for ML and/or a bipartition posterior probability of 0.95 or greater for the BI were regarded as sufficiently resolved (Huelsenbeck and Hillis 1993, Larget and Simon 1999). Pairwise (uncorrected-p) sequence distances were also calculated using MEGA 6.06 (Tamura et al. 2013).

Nucleotide sequences obtained in this study have been deposited in the GenBank database under the GenBank ID: KJ551848–KJ551855.

## Results

**Figure 2.**
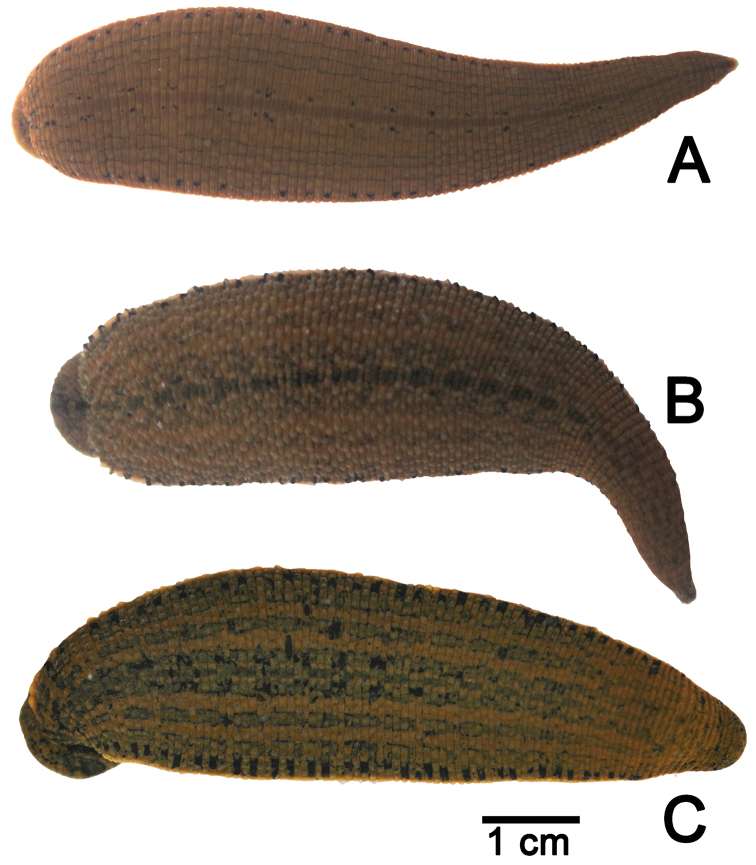
The color pattern of **A** *Hirudinaria javanica* CUMZ 3424 from Mukdahan **B** *Hirudinaria manillensis* CUMZ 3403 from Nakhon Phanom, and **C** *Hirudinaria* sp. CUMZ 3405 from Nakhon Phanom.

**Figure 3.**
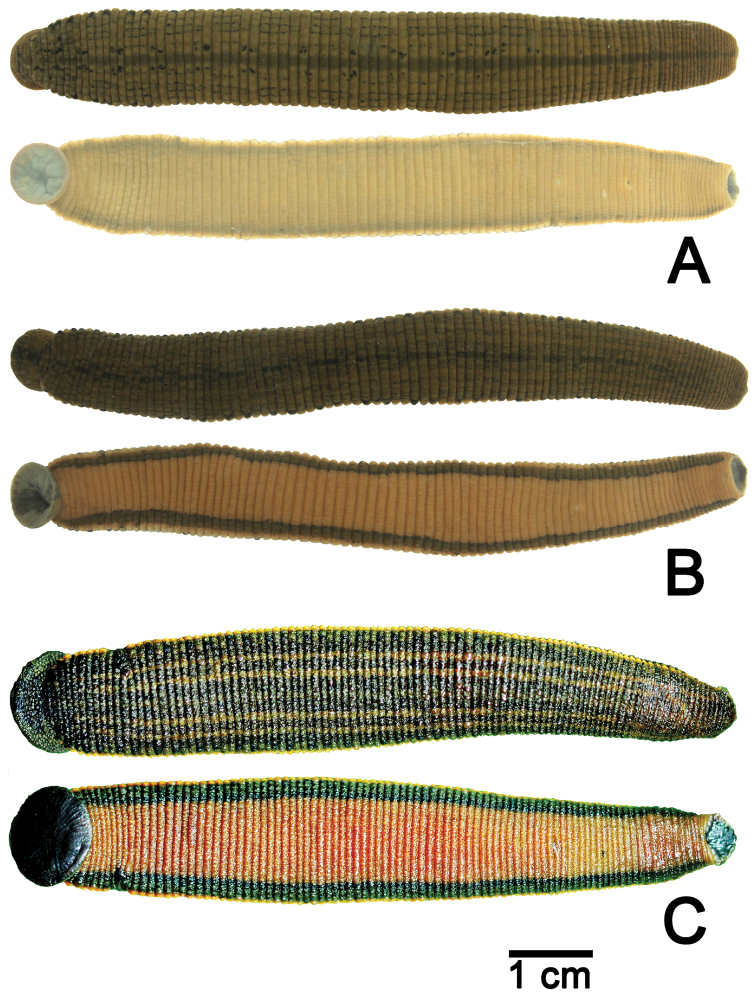
The dorsal and ventral sides of **A** *Hirudinaria javanica* CUMZ 3404 from Nakhon Phanom **B** *Hirudinaria manillensis* CUMZ 3403 from Nakhon Phanom, and **C** *Hirudinaria* sp. CUMZ 3406 from Nakhon Phanom.

All 435 examined specimens in this study were assigned as belonging to the genus *Hirudinaria* by the following distinct characters; male pore and female pore separated by 5–7 annuli, sensillae large and elongated, salivary papillae present, and without vaginal stalk. From these identified characters, the specimens were determined to be three species: as *Hirudinaria javanica*, *Hirudinaria manillensis*, and an unidentified morphotype, *Hirudinaria* sp. (Figs 2 and 3).

## Systematics

### Family Hirudinidae Whitman, 1886 Subfamily Hirudininae

#### Genus Hirudinaria

Taxon classificationAnimaliaArhynchobdellidaHirudinidae

Whitman, 1886

Hirudinaria Whitman, 1886: 373. Moore 1927: 207.

##### Type species.

*Sanguisuga javanica* Wahlberg, 1856, by original designation.

#### Hirudinaria javanica

Taxon classificationAnimaliaArhynchobdellidaHirudinidae

(Wahlberg, 1856)

**Figure 4.**
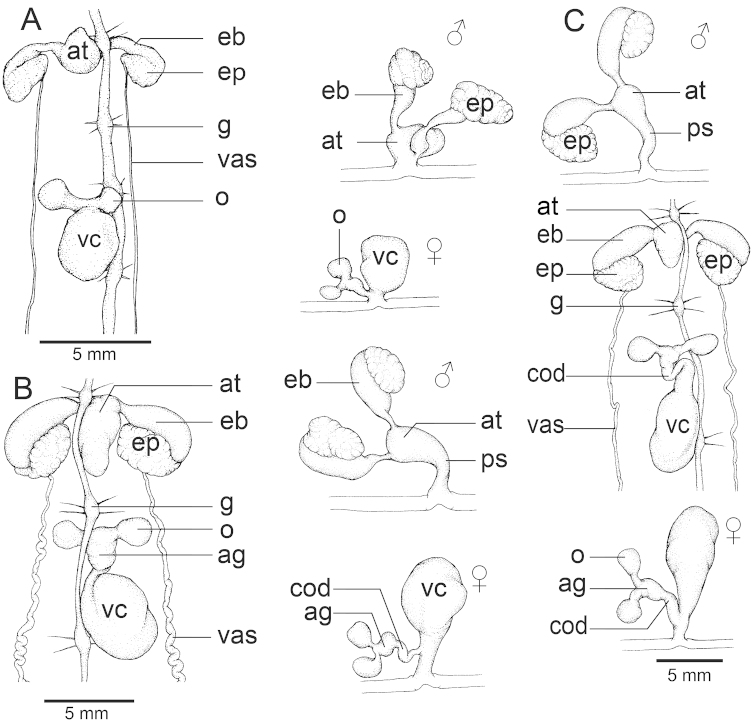
Illustrations of the reproductive system of **A** *Hirudinaria javanica* CUMZ 3404 from Nakhon Phanom, **B** *Hirudinaria manillensis* CUMZ 3403 from Nakhon Phanom, and **C** *Hirudinaria* sp. CUMZ 3405 from Nakhon Phanom. Abbreviations are: ag = albumin gland, at = atrium, cod = common oviduct, eb = ejaculatory bulb, ep = epididymis, g = ganglion, o = ovary, ps = penis sheath, vas = vas deferens, vc = vagina sac, vd = vagina duct.

**Figure 5.**
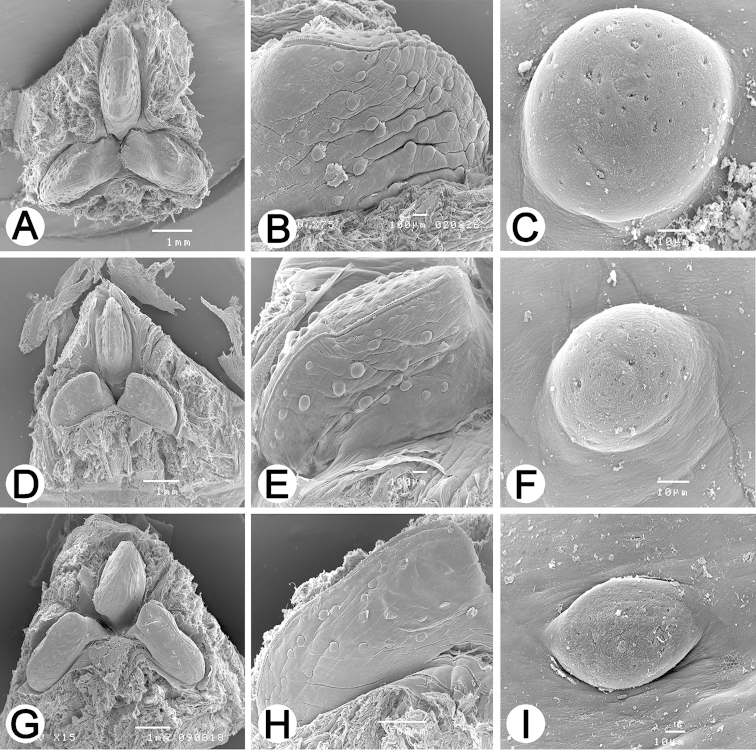
SEM images of the jaws of **A–C** *Hirudinaria javanica* CUMZ 3404 from Nakhon Phanom **D–F** *Hirudinaria manillensis* CUMZ 3403 from Nakhon Phanom, and **G–I** *Hirudinaria* sp. CUMZ 3405 from Nakhon Phanom. (**A, D, G**) overall jaw, (**B, E, H**) each jaw characteristic, and (**C, F, I**) salivary papillae.

Figs 2A , 3A , 4A , 5A–C

Sanguisuga javanica Wahlberg, 1856: 233. Type locality: Samarang, Java [Semarang, Central Java, Indonesia].
Hirudinaria javanica – Whitman 1886: 373–376, pl. 20, fig. 56.
Limnatis (Poecilobdella) javanica – Blanchard 1897: 349–351, text figure 7.
Limnatis javanica – Kaburaki 1921: 711.
Hirudinaria javanica – Moore 1927: 210–218, figs 50–52.

##### Material examined.

**Figure 6.**
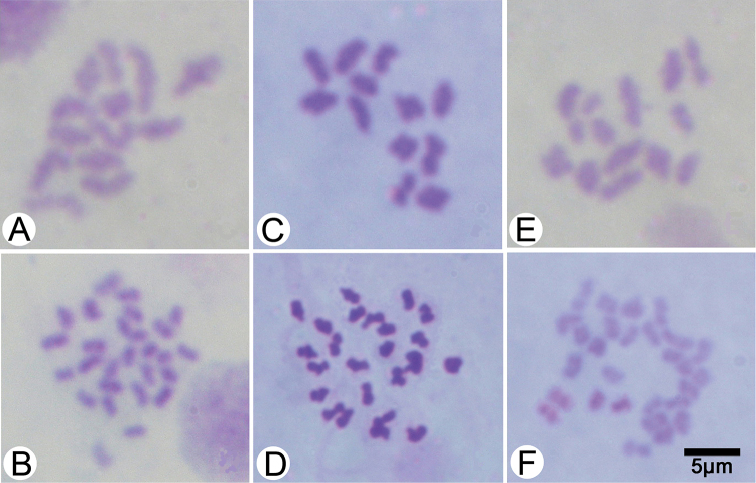
Meiotic and mitotic metaphase chromosome spreads of **A, B** *Hirudinaria javanica* (n = 13, 2n = 26) CUMZ 3424 from Mukdahan **C, D** *Hirudinaria manillensis* (n = 12, 2n = 24) CUMZ 3407 from Mahasarakham, and **E, F** *Hirudinaria* sp. (n = 14, 2n = 28) CUMZ 3406 from Nakhon Phanom.

Ban Donsala, Na Wa, Nakhon Phanom: CUMZ 3402 (17 specimens), 3404 (18 specimens; Figs 3A, 4A, 5A–C), 3429 (9 specimens). Ban Nongwang, Tao Ngoi, Sakon Nakhon: CUMZ 3413 (9 specimens). Ban Janpen, Tao Ngoi, Sakon Nakhon: CUMZ 3415 (16 specimens). Ban Nonghai, Khamchaee, Mukdahan: CUMZ 3422 (4 specimens), 3424 (9 specimens; Figs 2A, 6A–B). Chaturaphak Phiman, Roi Et: CUMZ 3419 (1specimen).

##### Description.

In preserved specimens body length 41–184 mm, width 5–16 mm. In live specimens, dorsal side olive green, dark green or yellow brown. Middle dorsal line distinct, black, continuous, parallel with two series of black spots on both sides, two faint black stripes present on each side. Body margin yellow with one ordered series of black spots. Ventral side green without marker. Jaw trignathous, approximately 134 teeth. Number of salivary papillae, both small and large, is 43 glands (Fig. 5A–C). Gonopores separated by seven annuli. Male reproductive system located in middle of body between somites XI and XIII. Ejaculatory bulbs short and small. Ejaculatory ducts long, connect with atrium side in somite XI. Atrium short, small, pear-shaped with unclear penis sheath. Vas deferens straight, runs along almost entire body, with 11 testisac pairs (12 pairs in some specimens). Nerve cord runs along body length on right side of atrium. Ovisacs stout, albumin gland not well developed, common oviduct short, opens into female bursa. Vagina caecum short, ovate in shape, no vaginal stalk (Fig. 4A).

#### Hirudinaria manillensis

Taxon classificationAnimaliaArhynchobdellidaHirudinidae

(Lesson, 1842)

Figs 2B , 3B , 4B , 5D–F

Hirudo manillensis Lesson, 1842: 8. Type locality: Philippine Islands.
Hirudo sanguisorba Tennent, 1859: 305. Type locality: Ceylon. Tennent 1861: 483–484, with text figure. Type locality: Caylon [Sri Lanka].
Hirudo multistriata Schmarda, 1861: 3, Taf. 16, fig. 141. Type locality: Ceylon [Sri Lanka].
Hirudo luzoniae Kinberg, 1866: 356. Type locality: Manila [Philippines].
Hirudo maculosa Grube, 1868: 39–40, Taf. 4, fig. 6. Type locality: Singapore.
Hirudo maculata Baird, 1869: 315. Type locality: Siam [Thailand].
Limnatis (Poecilobdella) granulosa Blanchard, 1893: 28. Type locality: Java, Indonesia Blanchard 1897: 338–349, figs 3–6. Kaburaki 1921: 673–675.
Limnatis granulosa – Robertson 1909: 676–679, fig. 4.
Hirudo boyntoni Wharton, 1913: 369–371. Type locality: Philippines Islands.
Limnatis maculosa – Dequal 1917: 9.
Limnatis (Poecilobdella) manillensis – Moore 1924: 376.
Hirudinaria manillensis – Moore 1927: 218–226, fig. 53.

##### Material examined.

Ban Donsala, Na Wa, Nakhon Phanom: CUMZ 3401 (21 specimens), 3403 (4 specimens; Figs 2B, 3B, 4B, 5D–F), 3430 (57 specimens). Ban Majang, Na Wa, Nakhon Phanom: CUMZ 3427 (51 specimens). Ban Nongwang, Tao Ngoi, Sakon Nakhon: CUMZ 3412 (4 specimens). Phang Khon, Sakon Nakhon: CUMZ 3428 (2 specimens). Mueang, Sakon Nakhon: CUMZ 3417 (2 specimens). Phu Phan, Sakon Nakhon: CUMZ 3416 (6 specimens). Ban Janpen, Tao Ngoi, Sakon Nakhon: CUMZ 3414 (1 specimen). Ban Nonghai, Khamchaee, Mukdahan: CUMZ 3423 (13 specimens). Khong Chai, Kalasin: CUMZ 3409 (28 specimens). Ban Thatoom, Mueang, Mahasarakham: CUMZ 3407 (4 specimens; Figs 6C–D). Huai E-pong, Phu Wiang, Khon Kaen: CUMZ 3425 (11 specimens). Bung, Mueang, Amnat Charoen: CUMZ 3410 (30 specimens). Pa Tio, Yasothon: CUMZ 3411 (3 specimens). Khemarat, Ubon Ratchathani: CUMZ 3408 (26 specimens). Chaturaphak Phiman, Roi Et: CUMZ 3418 (5 specimens). Kaset Wisai, Roi Et: CUMZ 3426 (67 specimens). Huai Saneng Reservoir, Surin: CUMZ 3420 (6 specimens), 3421 (5 specimens).

##### Description.

In preserved specimens, body length 27–248 mm, width 3–30 mm. In live specimens, dorsal side dark green or brown. Middle dorsal line distinct, black, incontinuous, with two faint black stripes on each side. Body margin yellow with disrupted black spots. Ventral side brown without marker. Jaw trignathous, approximately 148 teeth. Number of salivary papillae, both small and large sizes, is 30 glands (Fig. 5D–F). Gonopores separated by five annuli. Male reproductive system located in middle of body between somites XI and XII. Ejaculatory bulbs long and large. Ejaculatory ducts short, connect with atrium side in somite XI. Atrium relatively long, large, elongated in shape with penis sheath. Vas deferens curved, runs along almost entire body, 11 pairs of testisac. Nerve cord runs along body length on left side of atrium. Ovisacs stout, albumin gland well developed, common oviduct short, opening into female bursa. Vagina caecum relatively long, ovate in shape, no vaginal stalk (Fig. 4B).

#### Hirudinaria sp.

Taxon classificationAnimaliaArhynchobdellidaHirudinidae

Figs 2C , 3C , 4C , 5G–I

##### Material examined.

Ban Donsala, Na Wa, Nakhon Phanom: CUMZ 3405 (1 specimen; Figs 2C, 4C, 5G–I), 3431 (4 specimens). Ban Majang, Na Wa, Nakhon Phanom: CUMZ 3406 (1 specimen; Figs 3C, 6E–F).

##### Description.

In preserved specimens body length 107–140 mm, width 11–16 mm. In live specimens, dorsal side dark green, brown and dark brown. Middle dorsal line not present. Two brown stripes present each side of mid-dorsal region. Body margin yellow or orange with one ordered series of short black lines. Ventral side brown or dark brown without marker. Jaw trignathous, approximately 167 teeth. Number of salivary papillae, both small and large sizes, is 25 glands (Fig. 5G–I). Gonopores separated by five annuli. Male reproductive system located in middle of body between somites XI and XII. Ejaculatory ducts short, connect with atrium side in somite XI. Atrium moderate sized, penis sheath curved, opening on ventral side. Vas deferens relatively smooth, runs along almost entire body, 11 pairs of testis sacs. Nerve cord runs along body length on right atrium side. Ovisacs somewhat long, albumin gland well developed, common oviduct long, opening into female bursa. Vagina caecum long, elongated in shape, no vaginal stalk (Fig. 4C).

## Karyotype results

**Figure 7.**
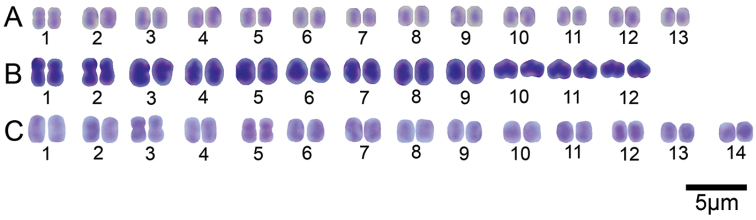
Karyotypes of **A** *Hirudinaria javanica* **B** *Hirudinaria manillensis*, and **C** *Hirudinaria* sp.

The chromosomes were typically indistinct because of their small size. Nevertheless, all cleared metaphase arrangements could be observed and the spermatogonial meiotic and mitotic chromosome numbers could be confirmed for all the examined species (Fig. 6). Haploid and diploid numbers of the three species of *Hirudinaria* were found to differ, ranging from n = 12, 2n = 24 for *Hirudinaria manillensis*, n = 13, 2n = 26 for *Hirudinaria javanica*, and n = 14, 2n = 28 for *Hirudinaria* sp. (Figs 6 and 7) and did not differ within each species across their respective geographic populations (Table 3). Chromosomal data of the three investigated *Hirudinaria* species obtained in the present study are summarized in Table 3 along with that for three other hirudinid species (all from the genus *Hirudo*) from the literature for comparison.

**Table 2.** Comparative morphological characters among *Hirudinaria* species in this study.

Characters	*Hirudinaria bpling*	*Hirudinaria javanica*	*Hirudinaria manillensis*	*Hirudinaria* sp.
Color	dark brown	dark green	dark brown/brown	dark green/brown
Distance (annuli) between male & female pores	5	7	5	5
Position of male and female organs	XI-XII	XI-XIII	XI-XII	XI-XII
Atrium	bulbous	short	long	relative long
Pairs of testisacs	-	12	11	11
Common oviduct	short	short	short	long
Vagina caecum	wide, long	small, ovate	small, ovate	large, elongate
References	Phillips (2012)	This study	This study	This study

**Table 3.** Comparison of chromosome numbers of the genera *Hirudo* and *Hirudinaria*.

Species	Locality no.^1^	No.^2^	Haploid (n)	Diploid (2n)	Reference
*Hirudo medicinalis*	Kharkiv, Ukraine	5	14	28	Utevsky et al. (2009)
*Hirudo verbana*	Odesa and Kharkiv, Ukraine	6	13	26	Utevsky et al. (2009)
*Hirudo orientalis*	Lake Taskul, Kazakhstan	7	12	24	Utevsky et al. (2009)
*Hirudinaria javanica*	1	4	13	26	This study
	3	1	13	26	This study
	7	2	13	26	This study
	8	3	13	26	This study
	15	1	13	26	This study
*Hirudinaria manillensis*	1	5	12	24	This study
	2	3	12	24	This study
	3	1	12	24	This study
	4	1	12	24	This study
	5	1	12	24	This study
	6	2	12	24	This study
	8	2	12	24	This study
	9	2	12	24	This study
	10	2	12	24	This study
	11	2	12	24	This study
	12	3	12	24	This study
	13	2	12	24	This study
	14	4	12	24	This study
	16	4	12	24	This study
	17	4	12	24	This study
*Hirudinaria* sp.	1	5	14	28	This study
	2	1	14	28	This study

1 Locality refers to the location where the sample was collected from, as coded in Table 1.

2 No = Number of specimens examined.

The karyotypes of all three species were asymmetric, and mostly telocentric, chromosomes. The distinct bi-armed chromosome marker varied among the three species, being found on the first pair in *Hirudinaria javanica*, on pairs 1, 2, 3 and 5 for *Hirudinaria manillensis* and on pairs 3 and 5 for *Hirudinaria* sp.

## Phylogenetic analysis

**Figure 8.**
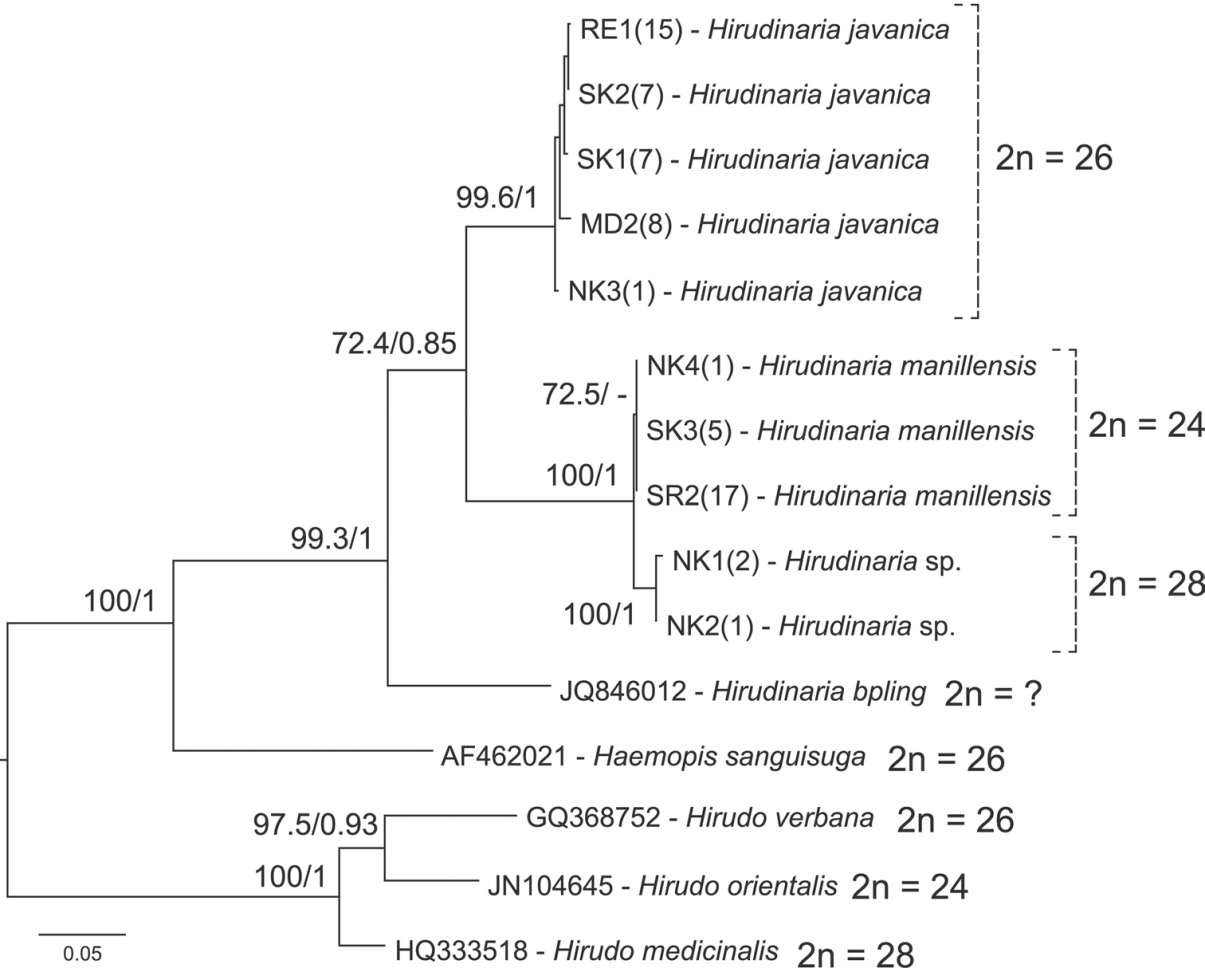
Phylogenetic relationships of the genus *Hirudinaria* and their related species, with chromosome number data. Tree topology was obtained from ML analysis based on a 658 bp fragment of the mitochondrial COI gene (DNA barcode region). Nodes with a 0.95 or higher bipartition posterior probability for BI and/or 70% or higher bootstrap value for ML were regarded as sufficiently resolved nodes, and are shown for the major clades (ML/BI). Numbers in parentheses refer to sampling localities in Figure 1 and the list in Table 1. Chromosome data of the related species were taken from Vitturi et al. (2002) and Utevsky et al. (2009).

The samples used for phylogenetic analysis and their collection locations are summarized in Table 4. A total of 3, 4 and 2 adult specimens of *Hirudinaria manillensis*, *Hirudinaria javanica* and *Hirudinaria* sp., respectively, were included. Fragments of the mitochondrial COI gene (DNA barcode region) containing 658 base pairs (bp) were used for the phylogenetic tree estimation. The final alignment data metric contained a total of 224 variable sites, 162 sites of which were parsimony informative. The nucleotide compositions of the gene fragments were A (28.32%), C (15.78%), G (15.51%) and T (40.39%). The phylogenetic tree showing the evolutionary relationships among *Hirudinaria* species and related taxa is shown in Fig. 8. Tree topology estimated by ML and BI analyses gave identical topologies with a high support for all major nodes (ML bootstrap values of 99.3–100% and a BI bipartition posterior probability of 1). The phylogenetic tree strongly supported the monophyly of the genus *Hirudinaria*. *Hirudinaria bpling* was basal to the *Hirudinaria javanica* and *Hirudinaria manillensis* clades. *Hirudinaria* sp. came out within the *Hirudinaria manillensis* clade.

**Table 4.** Taxa examined in the phylogenetic analysis, with collection localities and COI GenBank accession numbers.

Taxon	Locality no.^1^	Gen Bank accession nos.
*Hirudinaria javanica* (2n = 26)	1	KJ551852
	7	KJ551853, KJ551854
	8	KJ551851
	15	KJ551855
*Hirudinaria manillensis* (2n = 24)	1	KJ551850
	5	KJ551850
	17	KJ551850
*Hirudinaria* sp. (2n = 28)	1	KJ551848
	2	KJ551849
*Hirudinaria bpling*	Phang Nga, Thailand	JQ846012*
*Haemopis sanguisuga*	Sweden	AF462021*
*Hirudo verbana*	USA	GQ368752*
*Hirudo orientalis*	-	JN104645*
*Hirudo medicinalis*	Sweden	HQ333518*

1 Locality refers to the location where the sample was collected from, as coded in Table 1.

* Sequences were obtained from GenBank.

The uncorrected p-distances between the members of the genus *Hirudinaria* are shown in Table 5. The highest value of 0.132 was between *Hirudinaria bpling* and *Hirudinaria* sp. (2n = 28) and the lowest value of 0.014 was between *Hirudinaria manillensis* (2n = 24) and *Hirudinaria* sp. (2n = 28).

**Table 5.** Average uncorrected p-distance for the 658 bp COI gene sequences of the genus *Hirudinaria*.

Speceis	1	2	3	4
1. *Hirudinaria javanica* (2n = 26)	-			
2. *Hirudinaria manillensis* (2n = 24)	0.101	-		
3. *Hirudinaria* sp. (2n = 28)	0.110	0.014	-	
4. *Hirudinaria bpling*	0.119	0.129	0.132	-

## Discussion

All 435 examined specimens in this study were found by morphological analysis to belong to three distinct species within the genus *Hirudinaria*, and were identified as *Hirudinaria javanica*, *Hirudinaria manillensis* and an unidentified morphospecies (*Hirudinaria* sp.). They all shared various diagnostic characters reported in other studies, such as: a medium to large body size; five pairs of large eyes with the third and fourth pairs separated by one annulus, and the fourth and fifth pairs separated by two annuli; a large jaw; the presence of salivary papillae; gonopores separated by 5–7 annuli, and the absence of a vaginal stalk (Whitman 1886, Moore 1927, Klemm 1972, Nesemann and Sharma 2001, Lai and Chen 2010).

The unidentified species (*Hirudinaria* sp.) was different from the other two (*Hirudinaria javanica* and *Hirudinaria manillensis*) in both its morphology and also in its chromosome number and karyotype. Morphologically, *Hirudinaria* sp. had fewer salivary papillae (25) than the other two species (43 and 30 for *Hirudinaria javanica* and *Hirudinaria manillensis*, respectively) and a higher estimated number of teeth per jaw (167 versus 134 and 148 for *Hirudinaria javanica* and *Hirudinaria manillensis*, respectively) (Fig. 5). Although previous studies have reported a higher number of teeth for *Hirudinaria javanica* and *Hirudinaria manillensis* at 150 and 145, respectively (Moore 1927, Phillips 2012), than found in this study, these were still lower than that found for *Hirudinaria* sp. in this study. Comparison of all the taxonomic characters (Table 2) revealed that *Hirudinaria* sp. was quite similar to *Hirudinaria manillensis* in terms of having the gonopores separated by five annuli, but it differed in color pattern (Figs 2 and 3). However, the phylogenetic analysis, based upon the 658 bp of the mitochondrial COI gene sequence, placed *Hirudinaria* sp. in the same clade as *Hirudinaria manillensis*. Thus, they may represent recently sympatrically separated species. *Hirudinaria manillensis* was the most abundant and frequently found species in this study (346/435 or 79.5% of the collected specimens and found in all 17 sampled localities), compared to 83 (19%) specimens from five locations for *Hirudinaria javanica* and the seemingly rarer 6 samples (1.4%) from only two locations for unidentified *Hirudinaria* sp. Surprisingly, the northeastern Thailand population of *Hirudinaria manillensis* examined in this study showed a distinctly different internal morphology from that previously reported elsewhere. It contained a nerve cord running along on the left side of the atrium, instead of the right side as previously reported (Lai and Chen 2010), and also as found in *Hirudinaria javanica* and *Hirudinaria* sp. in this study.

With respect to the karyotypic analysis, the haploid and diploid chromosome numbers were similar to those reported previously in other genera of Hirudinidae (n = 14 in *Hirudo medicinalis*, n = 12 in *Hirudo orientalis* and n = 13 in *Hirudo verbena*) (Utevsky et al. 2009), but differed in chromosome structure and morphology. Moreover, distinctive karyotypic chromosome markers were presented, such as a distinct bi-arm chromosome that was only found on the first pair in *Hirudinaria javanica*, on pairs 1, 2, 3 and 5 in *Hirudinaria manillensis*, and on pairs 3 and 5 in *Hirudinaria* sp. That *Hirudinaria manillensis* showed the lowest chromosome number and the widest distribution across northeastern Thailand of the sampled species is of interest since, in general, it is believed that the original or ancestor species has the lowest chromosome number and is often the most common species (Sumner 2003). The unidentified species (*Hirudinaria* sp.) in this study had the same haploid and diploid chromosome numbers as *Hirudo medicinalis*, but their karyotypes were different (Utevsky et al. 2009) and their phylogenetic placement was markedly different, being placed in well-supported distinct clades, confirming that they are indeed separate biological species.

Our current identification of these 435 samples to three morphospecies (two nominal species and one unidentified morphospecies) was quite clear because of the distinct appearance of their external and internal organs, and was supported by the distinct chromosome numbers and karyotypes of the analyzed samples of each species. However, given the apparent variation between that reported here for, for example, *Hirudinaria manillensis* and that reported for the same nominal species elsewhere, indicates a need for further comparative studies utilizing type specimens and additional molecular analysis of these and congener species, for species confirmation and prior to any further systematic discussion and taxonomic re-classification. In particular, the potential recent sympatric speciation of *Hirudinaria manillensis* and *Hirudinaria* sp. requires further confirmation.

## Supplementary Material

XML Treatment for Hirudinaria

XML Treatment for Hirudinaria javanica

XML Treatment for Hirudinaria manillensis

XML Treatment for Hirudinaria sp.
